# Real-time biopsychosocial antecedents and correlates of functional neurological symptoms in daily life: A pilot remote monitoring technology study

**DOI:** 10.1016/j.psychres.2024.116247

**Published:** 2024-12

**Authors:** Susannah Pick, L.S. Merritt Millman, Jessica Davies, John Hodsoll, Biba Stanton, Anthony S. David, Mark J. Edwards, Laura H. Goldstein, Mitul A. Mehta, Timothy R. Nicholson, A.A.T.S. Reinders, Joel S. Winston, Trudie Chalder, Matthew Hotopf

**Affiliations:** a Institute of Psychiatry, Psychology & Neuroscience, King's College London, United Kingdom; b King's College Hospital NHS Foundation Trust, United Kingdom; c Instutite of Mental Health, University College London, United Kingdom; d South London & Maudsley NHS Foundation Trust, United Kingdom

**Keywords:** Functional neurological disorder, Non*epileptic seizures, Dissociative seizures, Wearable, Mobile, Ecological momentary assessment, Digital

## Abstract

Functional neurological symptom disorder (FNSD) is a neuropsychiatric diagnosis referring to symptoms resembling those of neurological disorders, occurring without causal neuropathology. FNSD has a complex biopsychosocial aetiology but its mechanisms are poorly understood. Remote monitoring technologies (RMT) could provide critical insights into functional neurological symptoms (FNS) in real-world contexts. We examined the feasibility and acceptability of a novel RMT protocol, to identify psychobiological correlates and antecedents of FNS in everyday life. Seventeen individuals with FNS (seizures/motor) and 17 healthy controls (HC) completed ecological momentary assessments (EMA) eight times daily for 1-week, reporting FNS severity, associated physical and psychological symptoms, and subjectively significant events. Sleep quality was reported daily. Physiological variables were measured using wearable Fitbit 5 devices. Multilevel modelling examined variables associated with FNS variability. Average EMA completion rates were good in both groups (≥80%). At week-level, the FNS group reported significantly greater subjective arousal, pain, fatigue, dissociation, negative affect, daily events, stressful events, and sleep duration, compared to HC. Objective sleep disturbance and duration, and resting heartrate, were also significantly greater in the FNS sample. FNS severity correlated significantly with daily events, affect, subjective arousal, pain, fatigue and sleep disturbance, at day- or within-day levels. Daily events and negative affect were the most prominent time-lagged predictors of within-day moment-to-moment FNS severity. RMTs are feasible and acceptable tools for investigation of FNS in real-world settings, revealing daily events and negative affect as possible triggers of FNS. Interventions targeting affective reactivity and regulation might be beneficial in this group. Larger-scale, longer-term RMT studies are needed in this population.

## Highlights

• Subjective functional neurological symptoms (FNS) were monitored for 7-days.
• Ecological momentary assessment tracked FNS and other subjective experiences.
• Wearables assessed objective physiological signals, including autonomic markers.
• Our novel remote monitoring protocol was feasible, acceptable and valid.
• Daily events and negative affect were robust temporal predictors of FNS severity.

## 1 Introduction

Functional neurological symptom disorder (FNSD, American Psychiatric Association, 2013), also known as dissociative neurological symptom disorder (World Health Organisation, 2022), is a common and often debilitating neuropsychiatric disorder that can manifest as motor, sensory, seizure and/or cognitive symptoms, in the absence of causal neurological pathology (Aybek and Perez, 2022; Stone et al., 2005). A diagnosis of FNSD is made when functional neurological symptoms (FNS) warrant medical evaluation and cause significant distress and disruption to the patient (American Psychiatric Association, 2013). FNSD is classified as a somatic symptom disorder in the Diagnostic and Statistical Manual of Mental Disorders – fifth edition, whereas it is conceptualised as a dissociative disorder in the International Classification of Diseases – eleventh revision (World Health Organisation, 2022); a discrepancy reflecting the current lack of consensus on the aetiological underpinnings of the disorder. FNSD is broadly understood to have multifaceted biopsychosocial causes, including exposure to acute and/or chronic psychosocial stressors, psychological vulnerabilities such as dissociative tendencies, post-traumatic symptoms, anxiety and depression, in addition to experiences of other physical health problems or significant injuries or accidents (Brown and Reuber, 2016). However, there is limited understanding of precisely how these varied aetiological factors culminate in clinical symptoms resembling those of neurological disorders.

The proximal antecedents and underlying mechanisms of FNS also remain incompletely understood. Patients report a variety of short-term FNS triggers, including physical exertion, sleep disruption, emotions, social and work-related stressors (Geroin et al., 2022; Jungilligens et al., 2020; Millman et al., 2024), although a proportion of patients with FNSD are unable to identify specific symptom triggers (Pick, Mellers and Goldstein, 2016a; Reuber et al., 2011). Previous clinical and experimental studies have shown altered emotional and interoceptive processing, elevated autonomic arousal and stress reactivity, and/or heightened dissociation in FNSD samples, with some preliminary evidence that they may serve as triggers for FNS occurrence or worsening (e.g., Geroin et al., 2022; Jungilligens et al., 2020; Koreki et al., 2020; Millman et al., 2024; Pick et al., 2019; Pick et al., 2016a, Pick et al., 2016b; Pick, Millman et al., 2024; Pick, Rojas-Aguiluz et al., 2020; Ricciardi et al., 2021). On this basis, several explanatory models have highlighted these processes as potential mechanisms underlying FNS (e.g., Goldstein and Mellers, 2006; Jungilligens et al., 2022; Pick et al., 2019; Roberts and Reuber, 2014). Our recent evidence-based explanatory model (Pick et al., 2019), for example, proposed that altered emotional processing and affective hyperarousal in this population, associated with excessive limbic, autonomic and HPA-axis activation, might lead to the short-term generation of FNS via disruption to neurocircuits involved in awareness, behavioural and cognitive control, and initiation of automatic action sequences.

Remote monitoring technologies (RMTs) may be optimal tools for investigating mechanisms in FNSD, due to their capability for tracking symptoms, alongside other relevant physical, environmental and psychological variables, with temporal precision and ecological validity (e.g., Siddi et al., 2023; Zhang et al., 2021a,b). Wearables and smartphone sensors can unobtrusively capture objective health-related data (e.g., activity, sleep, cardiorespiratory functioning) longitudinally with minimal patient-burden. Ecological momentary assessment (EMA), on the other hand, uses repeated subjective probes to monitor moment-to-moment variability in participants’ everyday experiences, often with electronic devices (e.g., smartphones). EMA can thus obtain experiential data with excellent temporal granularity, minimising retrospective reporting biases, and highlighting dynamic interactions between symptoms and other daily experiences (Shiffman, 2009; Shiffman et al., 2008).

RMTs are particularly well suited to investigating mechanisms in FNSD due to their potential for simultaneous measurement of subjective experiences and objective markers, which can diverge in FNSD (Adewusi et al., 2021; Pick et al., 2019). The prominent temporal variability in symptom types and severity often seen in FNS may not be adequately captured with standard rating tools (Nicholson et al., 2020; Pick, Anderson et al., 2020) and may be more amenable to investigation with RMTs. Nevertheless, there have been few studies using RMTs in FNSD, and none using EMA specifically.

Three previous studies examined aspects of functional tremor using combinations of daily diary symptom monitoring and wrist-worn actigraphy. One study reported a discordance between subjective daily diary reports (3 x daily) and objective tremor frequency in the functional tremor sample (*n* = 10) relative to a neurological control group (*n* = 8), across a 5-day sampling period (Parees et al., 2012); however, this was only partially replicated in a larger study covering a 1-month sampling period (5 x daily diary reporting, functional tremor *n* = 14; neurological controls *n* = 19) (Kramer et al. (2019). Kramer et al. (2021) also examined data to explore the temporal relationships between subjective negative affect and subjective and objective functional tremor severity, showing weak correlations between the two in both FNSD and neurological controls that did not differ between these groups.

Another study examined sleep quality over six days in patients with functional seizures (FS, *n* = 17) using actigraphy, alongside daily diaries (1 x daily) to monitor subjective sleep, FS occurrence, affect and dissociation (Mousa et al., 2021). This study provided evidence for poorer subjective and objective sleep quality in the FS group compared to healthy controls (*n* = 20) although, unexpectedly, better sleep quality (longer duration, fewer awakenings) was associated with an increased likelihood of FS occurrence the following day. Across the week, diary entries revealed elevated dissociation and reduced positive affect in the FS group compared to controls, although neither affect not dissociation significantly predicted the occurrence of seizures on the next day.

These initial reports suggest the potential value that RMTs might hold for revealing the day-to-day and within-day variability and features of FNS, their antecedents and/or the pathophysiological processes underlying them. However, there are no previous studies using EMA with FNSD samples and there is a need for larger-scale, more intensive RMT studies in this population, to examine a wider range of potential subjective and objective antecedents and correlates of FNS variability.

### 1.1 Aims & hypotheses

We piloted a novel RMT protocol using EMA alongside wearables to investigate a range of subjective, environmental and physiological symptom antecedents and correlates in participants with functional seizures (FS) and/or functional motor symptoms (FMS). The aims were to examine the acceptability and validity of the procedures, and to obtain data on the most prominent correlates and predictors of FNS severity. We sought to assess potential effect sizes to inform the required sample size for a larger, fully powered RMT investigation (Pick, David et al., 2024). In the present study, we tested several related hypotheses, based on existing evidence and explanatory models, outlined above, as follows:

1. Compared to healthy controls (HCs), the FNSD sample would display significantly elevated:
  a. Objective autonomic arousal (heartrate [HR], electrodermal activity [EDA]) (Paredes-Echeverri et al., 2022; Pick et al., 2019).
  b. Subjectively stressful daily events (Apazoglou et al., 2017; Testa et al., 2012; Tojek et al., 2000).
  c. Negative affect (Brown and Reuber, 2016; Pick, Mellers & Golstein, 2016a).
  d. Dissociative states (Campbell et al., 2023; Goldstein and Mellers, 2006; Pick, Mellers and Goldstein, 2017; Pick, Rojas-Aguiluz et al., 2020).
2. These elevated variables would correlate with and/or predict FNS severity ratings at the day- and/or within-day level (Campbell et al., 2023; Geroin et al., 2022; Goldstein and Mellers, 2006; Millman et al., 2024; Pick, Mellers and Goldstein, 2017; Pick et al., 2019; Pick, Rojas-Aguiluz et al., 2020; Pick, Millman et al., 2024).
3. Associations between subjective affect/arousal and HR/EDA would be weaker in the FNSD group compared to HCs (Pick et al., 2019; Pick, Millman et al., 2024).

Additional physical symptoms such as pain, fatigue and sleep disturbances are common in people with FNSD and may share underlying mechanisms with FNS (Carle-Toulemonde et al., 2023; Forejtova et al., 2023; Graham and Kyle, 2017; Mousa et al., 2021). A proportion of patients with FNSD also report that their FNS are triggered by some of these other physical symptoms and/or by physical exertion (e.g., Geroin et al., 2022; Millman et al., 2024; Jungilligens et al., 2020); however, these relationships have not yet been assessed systematically in patients’ daily lives, aside from the study exploring the impact of sleep quality on FS by Mousa et al. (2021). Thus, we tested some exploratory hypotheses, as follows:

• Elevated subjective ratings of pain, fatigue, and sleep disruption would be seen in the FNSD group compared to HCs, and these physical symptoms would be correlated with FNS severity (Millman et al., 2024).
• Greater objective sleep disturbance and higher physical activity levels would be correlated with FNS ratings (Geroin et al., 2022; Millman et al., 2024).

## 2 Methods

The study was conducted between July-October 2022, as the final component of a broader pilot project investigating psychobiological mechanisms in individuals with FNSD. A dedicated FNSD Patient and Carer Advisory Panel contributed to designing the study and the King's College London High Risk Research Ethics Committee (HR/DP-21/22–28,714) approved the project. Written informed consent was obtained according to the Declaration of Helsinki.

### 2.1 Participants

Participants with FS or FMS as their primary complaint were recruited through advertisement shared via FNSD charitable organisations (FND Hope UK, FND Action). The clinical status of each FNSD participant was confirmed with medical documentation (i.e., a copy of a letter from a relevant healthcare professional stating clearly the diagnosis of FNSD). The eligibility criteria can be found in Table 1. At baseline, participants with FS identified their most consistent premonitory pre-ictal symptom, whilst those with FMS identified their primary motor symptom. Participants reported on their predominant FNS throughout the sampling period (Box 1). Participants with FS were included if they experienced at least two seizures per month, to ensure that adequate data would be captured on potential triggers of the events during the sampling period.

**Table 1:** Eligibility criteria.

	**Functional neurological symptom disorder (FNSD)**	**Healthy controls**	**All participants**
**Inclusion criteria**	• Primary diagnosis of FNSD with motor symptoms and/or seizures. • Participants with functional seizures should experience ≥2 seizures/month, with premonitory symptoms/sensations.		• 18–65 years old. • Normal/corrected eyesight. • Fluency in English language.
**Exclusion criteria**	• Physical or cognitive symptoms or disability impairing ability to engage with the protocol (e.g., severe/constant tremor, bilateral upper limb paralysis, seizure frequency >10/day).	• Any active major physical or mental health disorder. • Current/history of functional neurological symptoms.	• Diagnosis of major neurological disorder (e.g., epilepsy, multiple sclerosis). • Diagnosis of major cardiovascular disorder. • Diagnosis of major psychiatric disorder (e.g., active psychosis, alcohol or substance dependence). • Medication that could significantly affect cognitive or cardiovascular functioning (e.g., beta-blockers, multiple/high-dose opiates).

**Box 1:** Ecological momentary assessment items.

	Items: Right now, at the present moment….
**Functional seizures**	• I am experiencing my primary seizure warning symptom. (Severity$: 1–7) • How much impact have my functional seizures had on me since the last prompt? (Impact: 1–7) • How many functional seizures have I experienced since the last prompt? (numerical response)
**Functional motor symptoms**	• I am experiencing my primary functional motor symptom. (Severity$: 1–7) • How much impact have my functional motor symptoms had on me since the last prompt? (Impact: 1–7)
**Dissociation – depersonalisation**	• I feel disconnected from my own body. (1–7) • l feel separated from what is happening to me, like an actor in a movie, or a robot. (1–7)
**Dissociation – derealisation**	• Things seem unreal to me, as if I am in a dream. (1–7) • It seems like I am looking at the world through a fog. (1–7)
**Dissociation – amnesia**	• I cannot account for things that have recently happened. (1–7) • I feel spaced out, and/or have lost track of what is going on. (1–7)
**Negative affect**	I feel (1–7): • Scared • Upset • Nervous • Ashamed • Irritable • Hostile
**Positive affect**	I feel (1–7): • Enthusiastic • Determined • Excited • Alert • Proud • Strong
**Pain**	I am in bodily pain. (1–7)
**Fatigue**	I feel tired. (1–7)
Arousal*	I feel bodily arousal. (1–7)
**Contextual items**	• Where am I? (Response options: Home; Workplace; College/university; Friend or relative's home; Healthcare setting; Outdoor public area; Indoor public area; On public transport; Using private transport; Other (insert brief description) • Who am I with? (Response options: On my own; With friends; With family; With friends and family; With pets; Other (insert brief description) • What was I doing before the prompt? (Response options: Resting; Working; Eating/drinking; Studying; Passive leisure activity (e.g., watching TV); Active leisure activity (e.g., sports, walking, socialising); Household chores; Travelling; Self-care; Other (insert brief description).
**Sleep**#	• Last night, my sleep was disrupted. (1–7) • How many hours did I sleep last night? (numerical response)
**Daily events**	• Have I experienced any significant events since the last prompt? (Yes/No) • What was the most significant event that happened since the last prompt? (free text) • This event was pleasant. (1–7) • This event was stressful. (1–7)

$ FNS severity = primary outcome.

⁎ Participants were instructed to report on bodily arousal reflecting markers of sympathetic/autonomic arousal, with examples provided (e.g. racing heart, sweating, dry mouth).

# Sleep quality items were delivered as a stand-alone prompt every morning at 8am and remained available until participants responded. Participants were instructed to complete the prompts without looking at the objective sleep data from the Fitbit device. For all ratings 1–7, 1=Not at all and 7=Extremely Daily subjective sleep quality ratings captured perceived sleep disruption (1–7, Not at all - Extremely) and estimated duration of sleep (hours).

Healthy control participants were recruited through external advertisement on social media and other websites (e.g., Facebook, Twitter, Gumtree). Healthy controls were sampled with the aim of matching for age and gender at group-level. Major neurological, cardiovascular and psychiatric disorders were excluded from both groups to avoid any confounding influences on the results, as were medications that could influence main dependent variables.

As this study was primarily aimed at assessing the feasibility and acceptability of the methods and estimating effect sizes for our larger project (Pick, David et al., 2024), a formal a-priori power analysis was not conducted, and the sample size was instead based on pragmatic factors.

### 2.2 Materials & measures

#### 2.2.1 Background measures

This sample completed a detailed clinical interview at baseline (First et al., 2015), online self-report questionnaires, neurocognitive testing, and several experimental tasks, all reported elsewhere (Millman et al., 2023; 2024; Pick, Millman et al., 2023, 2024).

#### 2.2.2 Ecological momentary assessment (EMA)

The RealLife Exp application (LifeData, LLC) delivered EMA probes via smartphones (https://www.lifedatacorp.com/) eight times daily (Box 1), on a pseudorandom schedule between 8am-10pm, with at least one hour between notifications. Most items were rated using a Likert-scale, where 1=Not at all and 7=Extremely, following previous EMA protocols (Myin-Germeys et al., 2003a, Myin-Germeys et al., 2003b).

Items on FNS, pain, fatigue, arousal and sleep were developed by the expert investigators, in collaboration with an FNSD Patient and Carer Advisory Panel. Affect and psychological dissociation were monitored with items adapted from validated scales (Bremner et al., 1998; Watson et al., 1988). Contextual questions were asked at each prompt regarding the participant's location, social environment and what they had been doing immediately prior to the notification.

At each prompt, participants were also asked to report any subjectively significant events that had occurred since the previous prompt, including psychological, social/environmental and physical factors. Participants were able to log events 24-hours/day. Explicit instructions were given to participants to log any events that they felt had affected them in some way, either positive or negative. If a significant event was endorsed, participants were then asked to provide a brief free text description of the event, without any potentially identifiable details. They also rated how stressful (1–7) and pleasant (1–7) each event was. These methods followed numerous clinical EMA studies (e.g., Glaser et al., 2006; Havermans et al., 2010; Myin-Germeys et al., 2003a,b).

#### 2.2.3 Physiological signal monitoring

Physiological data were acquired using the Fitbit Charge 5 wearable, which was selected due to its acceptability, unobtrusiveness, long battery life (Polhemus et al., 2020), water resistance, capacity for EDA measurement, and adequate measurement properties (e.g., Benedetti et al., 2021; de Zambotti et al., 2018; Fuller et al., 2020; Haghayegh et al., 2019; Irwin and Gary, 2022).

Each participant was provided with a Fitbit device along with log-in details for a unique, anonymised Fitbit account that had been set-up in advance by the immediate research team. Once logged into that account, participants could sync the Fitbit device to the associated Fitbit software. Participants were instructed directly to refuse any requests for access to data from their own personal devices. The Fitbit data were streamed directly to the Fitbit platform at each sync during the sampling period (participants were asked to sync daily). The research team downloaded the data from the Fitbit platform at the end of each participant's sampling period. Each anonymised Fitbit account was deleted permanently at the earliest opportunity after the participant's data had been retrieved and stored securely on King's College London servers.

Heartrate was acquired with the Fitbit's wrist-worn photoplethysmography (PPG) sensor, which samples HR in beats/minute (minimum resolution 5 s). Daily physical activity was quantified from accelerometer and PPG data (total daily minutes active). Nightly sleep quality was operationalised as the total duration of sleep nightly (minutes), in addition to sleep efficiency, calculated from the total sleep duration (minutes) divided by the total amount of time in bed (minutes). Total nightly awakenings operationalised objective sleep disturbance. All sleep variables were derived from accelerometer and PPG data.

Participants were asked to complete an ‘EDA scan’ with the Fitbit immediately following each EMA prompt, which uses finger-tip contact with wrist-based sensors to measure the number of EDA fluctuations in a given period (total scan time 1–2 min).

### 2.3 Procedures

Written consent was obtained before data collection commenced, including explicit consent for engagement with the RMT applications. Participants were supported in installing the smartphone applications, with training and instruction manuals provided.

Participants completed a start-up session using the RealLife Exp application and the EMA protocol commenced on the following day. At the end of sampling, participants underwent debriefing remotely, provided their feedback, and received a £50 digital shopping voucher.

### 2.4 Data processing and analysis

#### 2.4.1 Data preparation

**Fig. 1:**
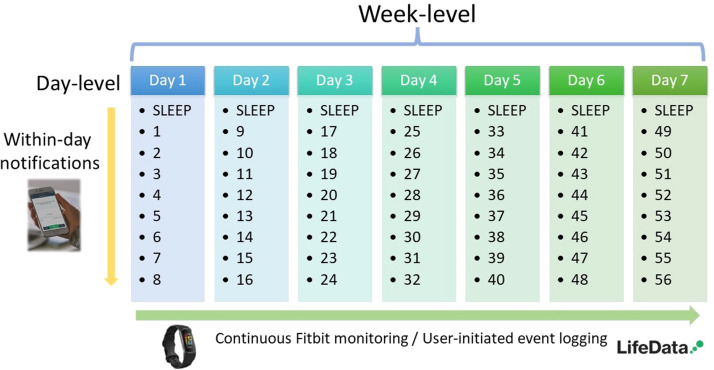
Remote monitoring datapoints.

The EMA data consisted of a maximum of 56 notification-initiated timepoints (level 1) × 17 participants (level 2) in each group (FNSD/HC), yielding 952 possible notification-initiated datapoints per group. EMA data were coded by timepoint (t: 1–56) and/or aggregated daily (d: 1–7), or at week-level. User-initiated data timepoints had no upper limit, as participants were able to log events at any time. Fig. 1 illustrates these timescales and datapoints.

Each reported daily event was coded as ‘not stressful’ or ‘stressful’ (0/1), and ‘not pleasant’ or ‘pleasant’ (0/1), using participants’ ratings on these dimensions (1–7; Not at all-Extremely). Events rated 1–3 (Not at all-Mildly) were coded as ‘not stressful’/’not pleasant’, whereas those rated 4–7 (Moderately-Extremely) were coded as ‘stressful’/’pleasant’. A total event count reflected all reported events at each prompt, as well as any user-initiated events reported between notification-initiated timepoints (duplicate event entries were excluded). Free text event descriptions were coded qualitatively into nine categories by two independent raters (LSMM, JD), with discrepancies resolved by a third rater (SP). FNS-related events were excluded from the counts for total, stressful, and pleasant events.

Mean daily resting HR was calculated from periods of inactivity during each 24-hour period, which were automatically detected by the Fitbit device on the basis of accelerometer data. HR values were extracted manually by the research team and computed for the 60-seconds associated with each EMA timepoint (t: 1–56), as well as the average values across each full 24-hour period (resting and active periods).

Mean EDA values were extracted from the first 10-seconds of each EDA scan (total EDA scans lasted from 1–2 min) manually by the research team based on timestamps which linked the EDA scan to the associated EMA timepoint (t: 1–56). EDA scan data were also aggregated by day (d: 1–7), and across the whole sampling period. To aggregate the data, average values were calculated for each participant by day (total number of day-level EDA responses/number of valid day-level scans) and by week (total number of week-level EDA responses/number of valid week-level scans). Week-level group means were calculated to reflect the average week-level EDA ‘lability’ of each group (i.e., the typical number of fluctuations per scan across participants in each group).

#### 2.4.2 Missing data and outliers

Rates of missing data were calculated for each variable by participant for the whole sampling period. As the primary purpose of this study was to assess feasibility, and multilevel modelling is suitable for use with missing data (Hox, 2017), we did not specify a cut-off for exclusion of cases based on missing data, nor did we impute missing values.

For aggregated, week-level scores, group-level outliers were identified as those individuals with scores above/below 2.5SD from their group mean. Sensitivity analyses examined the influence of group-level outliers at week-level. Participant-level outlying scores were included in the multilevel analyses because predictor variables were participant-centred and extreme scores on these variables may provide the most important insights into their relationships with FNS variability.

### 2.5 Statistical analyses

Statistical analyses were conducted in SPSS (IBM, v29) and ‘R’ (R Core Team, 2021). Statistical significance was assessed with alpha set at *p* ≤ 0.05. Benjamini-Hochberg adjustments (Benjamini and Hochberg, 1995) were applied to control the false discovery rate (20%).

#### 2.5.1 Sample **characteristics and week-level analyses**

Between-groups statistical tests compared the FNSD and HC groups on sociodemographic/clinical features and average weekly RMT variable values. Chi-squared/Fisher's exact (categorical), *t*-test (normal/continuous), or Mann-Whitney U (non-normal/continuous) tests were used as appropriate.

#### 2.5.2 Correlates and predictors of FNS severity

Multilevel linear mixed-effects regression models were generated as recommended for intensive longitudinal data (Bolger, 2013; Schwartz and Stone, 1998), to examine within-group (FNSD) relationships between RMT predictor variables and FNS severity ratings. Analyses were conducted with data aggregated at day and within-day (moment-to-moment) levels.

FNS severity was the outcome variable in all models. Timepoint (1–56) or day (1–7) were entered at level 1, nested within individual participants at level 2 (*n* = 17). All predictor variables were participant-centred (Bolger, 2013; Curran and Bauer, 2010). In all analyses, participant was entered as a random effect, with random intercepts in all models plus random slopes if the latter improved the model fit (Finch, Bolin and Kelly 2019). The chi-squared test of deviance and maximum likelihood estimation were used to compare models (Finch et al., 2019).

Concurrent analyses examined relationships between predictor variable values for a given timepoint (t) or day (d) and FNS severity values at the same timepoint/day (t, d). Time-lagged analyses assessed whether FNS ratings at a given timepoint (t) or on a given day (d) were predicted by scores on a predictor variable at the previous timepoint (t-1) or day (d-1) respectively.

Predictor variables that had significant relationships with FNS severity ratings were then entered into combined concurrent or time-lagged models to determine their relative influence. Relevant clinical and sociodemographic features were entered as covariates in the combined models, including baseline FNS severity scores, age, gender, and physical/mental health status.

#### 2.5.3 Relationships between objective and subjective arousal/affect

Concurrent multilevel models were generated with subjective arousal and negative/positive affect as outcome variables, with EMA-linked heartrate and EDA values as predictors, and participant entered as a random effect.

## 3 Results

### 3.1 Sample characteristics

Seventeen participants with FNSD with FS (*n* = 5) or FMS (*n* = 12) were compared to 17 HCs. Participants’ sociodemographic/clinical features are shown in Table 2. The self-report measure scores have been detailed previously (Millman et al., 2024; Supplementary Table 1).

**Table 2:** Sociodemographic and clinical features.

	FNSD (*n* = 17)	HC (*n* = 17)	Statistical values
**Age: M (SD)**	36.5 (10.6)	39.0 (11.0)	t(32)=0.67, *p* = 0.51
**Relationship status:** **n (%) married/cohabiting**	10 (59)	8 (47)	χ^2^=12, *p* = 0.73
**Ethnicity (% white)**	14 (82)	12 (71)	*p* = 0.688*
**Employment (% employed)**	2 (12)	13 (76)	*p* < 0.001*
**Education (% post-compulsory)**	16 (94)	17 (100)	*p* = 1.00*
**Gender (% female)**	13 (76)	13 (76)	*p* = 0.656*
**Medication (% yes)**	16 (94)	5 (29)	*p* < 0.001*
**Mental health diagnosis (% yes)**	10 (59)	1 (6)	*p* = 0.001*
**Physical health diagnosis (% yes)**	12 (71)	4 (24)	*p* = 0.007*

**Key:** FNSD=functional neurological symptom disorder; *M*=mean; SD=standard deviation.

⁎ Fisher's exact.

### 3.2 Feasibility

All participants completed the 7-day sampling period. The average EMA completion rate was 82% in the FNSD group and 80% in HCs. Wearable data were retrieved for 15 participants with FNSD (88%) and 16 HCs (94%), with data lost for three participants (FNSD=2, HCs=1) due to technical issues related to data syncing between the Fitbit device and platform (e.g., participant misunderstood instructions, failed to sync device, or accidentally deleted data).

Participants’ qualitative feedback indicated several factors that facilitated engagement with the study, including strong communication/technical support from the investigator, acceptability and user-friendliness of RMTs, relevance of tasks, and adequate/generous compensation. The EMA items were reported to be readily understandable, quick and convenient to complete. Some participants suggested reducing the intensity of the EMA schedule, adapting it to individual work and/or sleep patterns, and provision of video instructions for the apps/wearable. Most participants expressed willingness to participate in a longer sampling period, with reduced intensity.

### 3.3 Missing data & outliers

Average rates of missing data were in an acceptable range (<25%) for all EMA variables and most Fitbit variables (Supplementary Table 2). Completion rates for daily sleep ratings were excellent (>98%). The highest rates of missing data were for EDA in both groups (>30%) and EMA-linked HR (FNSD=26%, HCs=28%). There were no significant group differences in Fitbit wear-time or EMA response rates (Table 3). The total proportion of outlying datapoints was below 5% for all variables.

**Table 3:** Week-level group comparisons.

	FNSD (*n* = 17)*		HC (*n* = 17)*				
	M (SD)	Mdn (IQR)	M (SD)	Mdn (IQR)	Test statistic	p-value	Effect size
***Fitbit wear-time (days)***	6.6 (1.1)	7.0 (0.0)	6.9 (0.34)	7.0 (0.0)	*U* = 117.0	0.922	*r* = 0.04
***Percentage completed EMA responses***	82.3 (14.8)	87.0 (11.0)	79.7 (12.3)	81.0 (22.5)	*U* = 118.0	0.375	*r* = 0.16
***Arousal (1–7, weekly mean)***	2.9 (1.2)	3.0 (1.7)	1.5 (0.7)	1.2 (1.0)	*U* = 37.0	**<0.001**	*r* = 0.53
***Pain (1–7, weekly mean)***	3.2 (1.4)	3.2 (1.9)	1.33 (0.5)	1.2 (0.4)	*U* = 29.0	**<0.001**	*r* = 0.68
***Fatigue (1–7, weekly mean)***	4.1 (0.9)	4.3 (1.4)	2.8 (0.7)	2.9 (1.1)	*U* = 39.0	**<0.001**	*r* = 0.62
***Dissociation (1–7, weekly mean)***	1.9 (1.2)	1.6 (1.7)	1.1 (0.1)	1.0 (0.1)	*U* = 60.0	**0.003**	*r* = 0.50
***Positive affect (1–7, weekly mean)***	2.8 (1.1)	2.9 (2.0)	3.5 (1.4)	3.4 (1.7)	*U* = 109.0	0.231	*r*=−0.21
***Negative affect (1–7, weekly mean)***	1.6 (0.5)	1.5 (0.7)	1.3 (0.3)	1.1 (0.5)	*U* = 85.0	**0.041**	*r* = 0.35
***FNS severity (1–7, weekly mean)***	3.2 (1.2)	2.8 (1.1)	–	–	–	–	–
***FNSD impact***	3.0 (1.1)	2.8 (0.8)	–	–	–	–	–
***Subjective sleep disturbance (1–7, nightly mean)***	3.0 (1.0)	3.1 (1.6)	2.8 (1.1)	2.6 (1.1)	*U*= 124.5	0.496	*r* = 0.12
***Subjective sleep duration (nightly mean)***	7.3 (0.9)	7.0 (1.6)	6.4 (1.2)	6.6 (1.4)	*t*(32)=−2.37	**0.024**	*g* = 0.79
***Total seizure count (n = 5)***		18.0 (25.5)	–	–	–	–	–
***Total events (weekly)***		3.0 (4.5)		1.0 (2.0)	*U* = 67.0	**0.007**	*r* = 0.47
***Total stressful events (weekly)***		2.0 (3.0)		0.0 (1.0)	*U* = 85.0	**0.041**	*r* = 0.38
***Total pleasant events (weekly)***		0.0 (1.0)		0.0 (0.0)	*U* = 118.0	0.375	*r* = 0.20
***EDA (responses, weekly mean)***	28.1 (0.6)	28.0 (0.6)	28.5 (0.5)	28.5 (0.7)	t(29)= 1.94	**0.031**	*g*=−0.68
***Resting HR (BPM, daily mean)***	69.8 (7.1)	71.3 (10.0)	62.6 (6.6)	61.9 (8.3)	t(27)= −2.82	**0.004**	*g* = 1.02
***Total HR (BPM, daily mean)***	79.3 (6.8)	81.0 (6.8)	76.5 (7.3)	76.9 (9.2)	t(29)= −1.11	0.138	*g* = 0.39
***EMA-linked HR (BPM, weekly mean)***	84.5 (7.2)	85.8 (7.5)	81.4 (8.0)	79.8 (10.2)	t(29)= 1.12	0.137	*g* = 0.39
***Physical activity (mean mins/daily)***	233.5 (72.7)	233.3 (88.1)	266.8 (106.2)	286.4 (201.4)	t(29)= 1.01	0.320	*g*=−0.35
***Objective sleep duration*** ***(mean mins/night)***	433.0 (61.9)	450.0 (69.8)	386.6 (55.6)	395.4 (108.3)	t(29)= −2.20	**0.018**	*g* = 0.77
***Objective sleep disturbance (mean awakenings/night)***	29.3 (8.4)	31.3 (11.2)	22.7 (7.1)	20.6 (10.1)	t(20)= −2.36	**0.013**	*g* = 0.83
***Sleep efficiency (%; minutes asleep/minutes in bed)***	0.85 (0.06)	0.87 (0.03)	0.88 (0.04)	0.88 (0.03)	*U* = 78.0	0.102	*r*=−0.30

**Key:** BH=Benjamini-Hochberg; BPM=beats per minute; EDA=electrodermal activity; FNSD=functional neurological symptom disorder; HC=healthy controls; HR=heartrate; IQR=interquartile range; *M*=mean; Mdn=median; SD=standard deviation.

p-values in **bold** remained significant following Benjamini-Hochberg correction (20% false discovery rate).

⁎ Sample sizes for Fitbit variables were FNSD=15, HC=16 For all ratings 1–7, 1=Not at all and 7=Extremely.

EMA completion rates ranged from 60–100% in HCs and 49–100% in the FNSD group. Three participants in the FNSD group (61%, 52%, 49%) and five HCs (67%, 67%, 65%, 61%, 60%) completed less than 70% of EMAs.

### 3.4 Week-level analyses

Average week-level EMA scores were significantly elevated in the FNSD group versus HCs for arousal, pain, fatigue, dissociation, negative affect, sleep duration, total events, and stressful events (Table 3). There were significant between-group differences for average weekly EDA, resting heartrate, objective sleep duration and sleep disturbance. Significant results withstood Benjamini-Hochberg correction (Supplementary Table 3). Supplementary Table 4 presents the week-level results with outliers removed. The group difference for total stressful life events was no longer significant when one outlier was removed.

Contextual information linked to all notifications across the week sampling period can be found in Table 4. There were significant between-group differences on all three contextual items.

**Table 4:** Contextual details – notification-linked.

	FNSD (*n* = 17)	HC (*n* = 17)	Statistical values
	N (%)	N (%)	
**Total responses to notifications**	770	745	
**Item 1. Where am I?**			χ^2^=96.98, df=9, *p* < 0.001, Cramer's *V* = 0.25
***Home***	551 (71.6)	474 (63.6)	
***Workplace***	35 (4.5)	65 (8.7)	
***College/university***	7 (0.9)	8 (1.1)	
***Friend or relative's home***	38 (4.9)	3 (0.4)	
***Healthcare setting***	6 (0.8)	0 (0.0)	
***Outdoor public area***	37 (4.8)	100 (13.4)	
***Indoor public area***	41 (5.3)	40 (5.4)	
***On public transport***	10 (1.3)	22 (3.0)	
***Using private transport***	37 (4.8)	15 (2.0)	
***Other***	8 (1.0)	18 (2.4)	
**Item 2. Who am I with?**			χ^2^=52.66, df=4, *p* < 0.001, Cramer's *V* = 0.19
***On my own***	289 (37.6)	373 (50.0)	
***With friends***	53 (6.9)	75 (10.1)	
***With family***	341 (44.3)	264 (35.5)	
***With pets***	36 (4.7)	6 (0.8)	
***Other***	51 (6.6)	27 (3.6)	
Item 3. What was I doing immediately before?*			χ^2^=91.83, df=9, *p* < 0.001, Cramer's *V* = 0.25
***Resting***	168 (21.9)	100 (13.4)	
***Working***	55 (7.2)	129 (17.3)	
***Eating/drinking***	78 (10.2)	94 (12.6)	
***Studying***	8 (1.0)	27 (3.6)	
***Passive leisure activity***	192 (25.0)	122 (16.4)	
***Active leisure activity***	70 (9.1)	89 (12.0)	
***Household chores***	57 (7.4)	87 (11.7)	
***Travelling***	59 (7.7)	42 (5.6)	
***Self-care***	34 (4.4)	18 (2.4)	
***Other***	47 (6.1)	36 (4.8)	

*FNSD *n* = 768, HC *n* = 744.

Details regarding the types of events reported are presented in Table 5. There was a significant between-group difference in the types of events reported, with FNS-related events excluded (χ2=17.47, df=7, *p* = 0.01, Cramer's *V* = 0.42).

**Table 5:** Significant events: type and context.

	FNSD (*n* = 17)	HC (*n* = 17)
	N (%)	N (%)
**Total significant events reported**	100	23
**Event type**		
***FNS***	26 (26.0)	–
***Physical health***	23 (23.0)	4 (17.4)
***Sensory stimulation***	6 (6.0)	0 (0.0)
***Social conflict***	10 (10.0)	1 (4.3)
***Physical activity/exercise***	5 (5.0)	2 (8.7)
***Emotional event/situation***	5 (5.0)	5 (21.7)
***Leisure activity***	9 (9.0)	4 (17.4)
***Social event/activity***	1 (1.0)	4 (17.4)
***Combination of above/other***	15 (15.0)	3 (13.0)

### 3.5 Day-level analyses

Table 6 presents statistical values for associations between day-level predictor variables and day-level FNS severity. Concurrent multilevel models indicated that the following variables were positively associated with day-level FNS severity ratings: subjective arousal, pain, fatigue, positive (inverse) and negative affect, EMA-linked heartrate, objective sleep disturbance (awakenings), total events, stressful events, and pleasant events. All associations withstood Benjamini-Hochberg correction (Supplementary Table 5). None of the possible predictors were significant in the day-level time-lagged analyses.

**Table 6:** Day-level relationships between each predictor variable and FNS severity.

	Concurrent				Time-Lagged			
	*ß*	*SE*	95% CI	p-value	*ß*	*SE*	95% CI	p-value
***Subjective arousal***	0.478	0.133	0.199 - 0.757	**0.002**	−0.114	0.106	−0.324 - 0.095	0.281
***Pain***	0.567	0.134	0.281 - 0.853	**<0.001**	0.086	0.118	−0.148 - 0.320	0.467
***Fatigue***	0.444	0.095	0.256 - 0.632	**<0.001**	−0.049	0.116	−0.280 - 0.182	0.675
***Dissociation***	0.381	0.330	−0.360 – 1.12	0.277	0.128	0.205	−0.279 - 0.536	0.533
***Positive affect***	−0.392	0.126	−0.641 – −0.142	**0.002**	0.122	0.158	−0.192 - 0.435	0.443
***Negative affect***	0.575	0.175	0.227 - 0.923	**0.001**	−0.342	0.198	−0.736 – 0.051	0.087
***Subjective sleep disturbance***	0.046	0.050	−0.054 - 0.147	0.360	0.036	0.062	−0.088 −0.159	0.568
***Subjective sleep duration***	−0.004	0.062	−0.128 - 0.119	0.947	0.092	0.072	−0.051 - 0.235	0.206
***Total events***	0.283	0.083	0.119 – 0.447	**<0.001**	−0.097	0.097	−0.290 - 0.096	0.319
***Stressful events***	0.431	0.153	0.127 – 0.735	**0.006**	−0.114	0.167	−0.447 - 0.218	0.496
***Pleasant events***	0.311	0.126	0.061 – 0.562	**0.015**	−0.132	0.141	−0.413 – 0.149	0.354
***EDA***	0.036	0.326	−0.612 – 0.685	0.912	−0.603	0.691	−1.978 - 0.772	0.386
***Resting HR (BPM)***	−0.025	0.049	−0.122 - 0.072	0.608	−0.008	0.052	−0.112 - 0.095	0.873
***Total HR (BPM)***	0.007	0.022	- 0.037 - 0.052	0.740	0.036	0.034	−0.033 - 0.104	0.301
***EMA-linked HR (BPM)***	0.029	0.014	0.001 - 0.058	**0.045**	−0.003	0.020	−0.053 – 0.046	0.874
***Physical activity*** ***(mins)***	−0.001	0.001	−0.003 - 0.001	0.474	0.002	0.001	0.00 – 0.004	0.117
***Objective sleep duration*** ***(mins)***	−0.001	0.001	−0.002 - 0.000	0.126	0.000	0.001	−0.001 - 0.002	0.574
***Objective sleep disturbance (awakenings)***	0.015	0.007	0.001 - 0.029	**0.033**	0.007	0.009	−0.011 - 0.025	0.442

**Key:** BPM=beats per minute; CI=confidence interval; *d*=day; EDA=electrodermal activity; EMA=ecological momentary assessment; HR=heartrate; SE=standard error.

p-values in **bold** were significant following Benjamini-Hochberg correction.

When combined in a single concurrent model (Supplementary Table 6), the predictors remaining significant were pain (*ß*=0.322, *p* = 0.002), positive affect (*ß*=−0.222, *p* = 0.046) and total daily events (*ß*=0.226, *p* < 0.001). After controlling for relevant covariates, subjective arousal (*ß*=0.215, *p* = 0.041), pain (*ß*=0.302, *p* = 0.002), fatigue (*ß*=0.294, *p* = 0.019), total daily events (*ß*=0.187, *p* = 0.010), and EMA-linked HR (*ß*=0.018, *p* = 0.048) were significant.

Supplementary Figure 1 presents the day-level standardised EMA variable ratings in the FNSD group, at the participant level (Red=Higher than participant’s mean; Green=Lower than participant’s mean; Black=Missing data).

### 3.6 Within-day (momentary) analyses

Table 7 presents the statistical values for the multilevel models assessing within-day associations between all predictor variables and FNS severity ratings. Significant correlates of momentary FNS severity in concurrent models were subjective arousal, pain, fatigue, positive affect, negative affect, total events and stressful events. Pain, fatigue, positive affect, negative affect, dissociation and total events were significant predictors in the time-lagged models. EDA and EMA-linked HR were not significant predictors of momentary FNS severity. The overall pattern of results remained the same after Benjamini-Hochberg correction (Supplementary Table 7).

**Table 7:** Momentary relationships between each predictor variable and FNS severity.

	Concurrent				Time-Lagged			
	*ß*	*SE*	95% CI	p-value	*ß*	*SE*	95% CI	p-value
***Subjective arousal***	0.244	0.074	0.088 - 0.400	**0.004**	0.103	0.060	−0.023 - 0.229	0.103
***Pain***	0.496	0.094	0.292 – 0.700	**<0.001**	0.195	0.077	0.030 - 0.361	**0.023**
***Fatigue***	0.341	0.065	0.205 – 0.477	**<0.001**	0.107	0.042	0.025 - 0.190	**0.011**
***Dissociation***	0.716	0.385	−0.565 – 1.99	0.167	0.158	0.066	0.029 - 0.287	**0.016**
***Positive affect***	−0.334	0.088	−0.520 - −0.148	**0.002**	−0.128	0.061	−0.248 - −0.008	**0.037**
***Negative affect***	0.541	0.114	0.280 – 0.803	**0.001**	0.227	0.082	0.065 – 0.389	**0.006**
***Total events***	1.00	0.194	0.529 – 1.48	**0.002**	0.534	0.175	0.192 – 0.877	**0.002**
***Stressful events***	1.12	0.187	0.754 – 1.49	**<0.001**	0.419	0.214	−0.002 – 0.840	0.051
***Pleasant events***	0.138	0.291	−0.433 – 0.709	0.635	0.538	0.348	−0.145 – 1.22	0.123
***EDA***	0.036	0.114	−0.188 – 0.261	0.750	0.011	0.131	−0.247 - 0.269	0.931
***EMA-linked HR (BPM)***	0.001	0.004	−0.007 - 0.009	0.767	0.006	0.005	−0.003 - 0.015	0.174

**Key:** BPM=beats per minute; CI=confidence interval; EDA=electrodermal activity; EMA=ecological momentary assessment; HR=heartrate; SE=standard error.

p-values in **bold** remained significant following Benjamini-Hochberg correction.

Supplementary Figure 2 displays the within-day EMA variable ratings in the FNSD group, at the participant level (Red=Higher than participant’s mean; Green=Lower than participant’s mean; Black=Missing data).

When the significant concurrent correlates of momentary FNS severity were entered in a combined model (Supplementary Table 8), pain (*ß*=0.325, *p* < 0.001), fatigue (*ß*=0.158, *p* < 0.001), positive affect (*ß*=−0.104, *p* = 0.023) and total events (*ß*=0.609, *p* < 0.001) remained significant. After controlling for covariates, the same variables were significant (p-values 0.002–0.044), except positive affect (*p* = 0.322).

A final model including the significant time-lagged predictors (Supplementary Table 9) resulted in none of the predictors reaching significance. In the covariate-adjusted model, only negative affect significantly predicted FNS severity (*ß*=0.239, *p* = 0.035).

### 3.7 Objective and subjective arousal and affect

#### 3.7.1 EDA as predictor of subjective arousal and affect

EDA did not predict momentary subjective arousal in HCs (*ß*=−0.144, *p* = 0.092); however, it did in the FNSD group (*ß*=−0.529, *p* = 0.036).

With positive affect as the outcome, EDA was a significant predictor in HCs (*ß*=−0.184, *p* = 0.015), but not in FNSD (*ß*=−0.23, *p* = 0.810). For negative affect, EDA was non-significant for both HCs (*ß*=−0.032, *p* = 0.255) and the FNSD group (*ß*=−0.083, *p* = 0.169). The significant results withstood Benjamini-Hochberg correction.

#### 3.7.2 Heartrate as predictor of concurrent subjective arousal and affect

EMA-linked heartrate predicted subjective arousal in HCs (*ß*=0.006, *p* = 0.007), but not in FNSD (*ß*=0.007, *p* = 0.109). For positive affect, EMA-linked heartrate was a significant predictor in both HCs (*ß*=0.007, *p* = 0.006) and FNSD (*ß*=0.008, *p* = 0.042). In contrast, EMA-linked heartrate did not predict negative affect in either HCs (*ß*=0.000, *p* = 0.658) or FNSD (*ß*=0.001, *p* = 0.622). Again, results remained significant after Benjamini-Hochberg correction.

## 4 Discussion

The aims of this study were to pilot a novel RMT protocol in FNSD and to identify biopsychosocial antecedents and correlates of FNS in patients’ daily lives. The protocol was valid and acceptable to participants. Notable antecedents of FNS were daily events and negative affective states, whereas correlates of FNS included pain, fatigue, subjective arousal and negative affect. Inconsistent results were observed between groups in the relationships between objective autonomic markers and subjective arousal/affect.

### 4.1 Feasibility

All enrolled participants completed the study, with good overall EMA response rates in both groups, although the range was greater in the FNSD group. Five participants in the HC group showed suboptimal compliance (completion of <70% prompts); however, three participants in the FNSD group also showed poor compliance (49–61% completion). Nevertheless, there was good overall adherence to wearing the Fitbit across groups. Participants’ feedback highlighted the importance of strong technical support and communication with the investigator, adequate compensation, and the user-friendliness of the smartphone apps/wearable. Suggestions for improvement focused on extending the monitoring period and reducing the EMA intensity. As such, individuals with FNSD readily engaged with this RMT study, providing a promising basis for future investigations.

### 4.2 Week-level between-group differences

#### 4.2.1 Subjective symptoms

Across the 7-day monitoring period, this FNSD sample experienced elevated subjective physiological arousal, pain, fatigue, negative affect and dissociation, compared to HCs. These findings are consistent with our hypotheses and other studies that utilised retrospective self-report questionnaires in other FNSD samples (Brown and Reuber, 2016; Campbell et al., 2023; Carle-Toulemonde et al., 2023; Forejtova et al., 2023; Goldstein and Mellers, 2006; Pick, Mellers and Goldstein, 2017). These results also accord with findings from the validated questionnaire data obtained with the present sample, in which they reported heightened self-reported general somatic symptoms, depression/anxiety, and dissociation (Millman et al., 2024). In our related laboratory-based study of affective reactivity with this sample (Pick, Millman et al., 2024, momentary reports of pain, fatigue and dissociative amnesia were elevated during baseline and the affective picture viewing task in the FNSD sample compred to controls, consistently with the present remote monitoring findings. However, in the laboratory study, momentary subjective physiological arousal, affect, depersonalisation and derealisation were not elevated significantly in this FNSD sample at baseline or during the task (Pick, Millman et al., 2024). These intriguing differences in the remote and laboratory-based results require further examination in larger studies.

#### 4.2.2 Daily events

This FNSD sample reported a greater number of total and stressful daily events than HCs (encompassing stressful and stressful/pleasant events), similarly to previous studies showing elevated stressful event reports in patients with FS using retrospective measures (e.g., Testa et al., 2012; Tojek et al., 2000). The qualitative descriptions of daily events showed that, aside from FNS-related instances, the most frequent events reported by the FNSD group were physical health issues, social conflict, and ‘Combination/other’ events. In the HC group, emotional events were the most common, followed by physical health, leisure activities, and social events. Further examination of the nature of daily events (e.g., hassles/uplifts) affecting people with FNSD in comparison to control groups seems warranted.

#### 4.2.3 Contextual factors

The contextual data sampled pseudorandomly alongside each notification showed that the FNSD group differed significantly to HCs in their typical reported location, social context, and day-to-day activities. The FNSD sample tended to spend more time in the home and less time in outdoor public spaces or in the workplace than HCs, which could be due to physical and/or psychological factors linked to FNSD that can present challenges to leaving a home-based environment and/or engaging in employment or activities in public spaces (Dosanjh et al., 2021; Ranford et al., 2020; Wyatt et al., 2014). While FNSD participants spent more time with family members and pets, HCs were more often with friends or on their own. The significant time that FNSD participants spent with family members is unsurprising, as several studies have noted the care and support that family members can provide (Dickinson et al., 2011; Rawlings et al., 2017). FNSD can make it difficult to engage in activities independently, and thus HCs may have reported significantly more time on their own due to a greater degree of autonomy and independence. In addition, HCs spent significantly more time with friends than the FNSD group, possibly reflecting the marked impact that FNSD can have on social functioning. There were also group differences in the activities that participants reported immediately prior to the prompts, with the FNSD group being more likely to be resting or engaging in passive leisure activities than HCs, probably related to the emotional and physical limitations caused by FNS and associated fatigue (Fairclough et al., 2014; Rawlings et al., 2017). Together, these contextual data demonstrate the substantial impact that FNSD can have on patients’ everyday lives, also indicating the potential value of remote monitoring technologies for assessing important clinical outcomes in this population.

#### 4.2.4 Objective physiological variables

The week-level Fitbit data demonstrated that the FNSD group exhibited extended nightly sleep duration and more disturbed sleep, but similar sleep efficiency scores, compared to HCs. Our findings partially concurred with Mousa et al. (2021) who also observed more objective sleep disturbances in an FS sample. In summary, our data indicated that sleep quality rather than quantity was most disrupted in this sample, but further studies are needed to assess objective and subjective sleep in FNSD on a longer-term basis.

The week-level observation of fewer EDA responses in the FNSD group is inconsistent with some previous reports of elevated autonomic activation in some FNSD samples (Paredes-Echeverri et al., 2022). The HR findings were similarly mixed, showing elevated resting HR in the FNSD sample versus HCs, but no group differences in total HR (spanning resting and active periods) or EMA-linked HR. We note that whilst not measured here, lower respiratory sinus arrhythmia (a measure of heartrate variability) has previously been seen in this population (Roberts et al., 2012), which can overlap with higher baseline/resting heartrate. Furthermore, in previous studies, heartrate reactivity (active/responsive) has been found to differ between groups less often than baseline differences (Paredes-Echeverri et al., 2022), so the lack of differences in total HR seen here (resting plus active) is not atypical.

Together, the EDA and HR findings presented here provide only limited demonstration of autonomic hyperactivation in the FNSD sample and some limitations of the present data should be recognised. Heightened resting state HR in the FNSD group, for example, could possibly be explained by general health status, although the groups were matched for BMI and age, they did not show significant differences in physical activity, and we had excluded cardiovascular disorders and related medications from both groups. These EDA/HR findings should nonetheless be interpreted with caution because there were high rates of missing data and the accuracy of the Fitbit EDA/HR sensors are unlikely to be akin to that of research-grade wearables or gold-standard methods (e.g., electrocardiography, Ag-AgCl EDA electrodes).

### 4.3 Correlates and predictors of fns severity ratings

#### 4.3.1 Daily events

Subjectively salient daily events (total, stressful, pleasant) were correlated positively with FNS severity in day-level and/or within-day analyses. Notably, salient events at a given timepoint were associated with increased FNS severity at the subsequent timepoint. These findings remained significant when relevant covariates were controlled for.

These results corroborate patients’ retrospective reports that daily events, including positive experiences and/or stressors, can trigger or exacerbate FNS (Geroin et al., 2022; Millman et al., 2024). Furthermore, these findings concur with our recent experimental observation that exposure to highly arousing affective images resulted in elevated subjective momentary FNS (Pick, Millman et al., 2024). Thus, emotionally arousing daily events appear to exert an aggravating influence on subjective FNS, in line with our current model (Pick et al., 2019). Together, these findings support the potential value of interventions directly targeting reactivity to emotionally salient events in everyday life.

#### 4.3.2 Affect

At the day and within-day levels, reduced positive affect and greater negative affect were associated with elevated FNS severity. The within-day analyses also showed that positive and negative affect predicted FNS severity from one timepoint to the next, although only negative affect remained significant after controlling for possible confounds.

The concurrent relationships between affect and FNS severity could reflect causal dynamics in which affective states exert an influence on FNS severity, FNS severity influences affective states, or both (Millman et al., 2024; Pick, Mellers and Goldstein, 2016a). However, the observation that heightened momentary negative affect predicted worse subsequent momentary FNS severity provides support for the proposal that negative emotional states might directly exacerbate or precipitate FNS occurrence in the short-term. These findings support a role for altered affective processing as a mechanism in FNSD (Goldstein and Mellers, 2006; Pick et al., 2016a, Pick et al., 2016b; 2018a,b; Pick et al., 2019; Pick, Millman et al., 2024), although one previous RMT study did not provide strong evidence for this (Kramer et al., 2021). Treatments aiming to improve awareness and regulation of negative affect on a day-to-day basis may be of considerable benefit to people with FNSD.

#### 4.3.3 Dissociation

Dissociation ratings were not correlated with FNS severity in concurrent analyses. Whilst the time-lagged analyses demonstrated that momentary dissociation predicted subsequent FNS ratings, this did not survive correction for possible confounds. These results do not offer persuasive evidence for an independent relationship between state dissociation and momentary FNS severity, contrary to previous studies and perspectives (Brown, 2002; Brown et al., 2007; Campbell et al., 2023; Goldstein and Mellers, 2006; Pick, Mellers and Goldstein, 2017; Pick, Rojas-Aguiluz et al., 2020). Here, we analysed dissociation as a composite score, incorporating depersonalisation, derealisation and amnesia, but specific types of dissociation might precede the onset or exacerbation of FNS. Dissociation might also be a particularly prominent trigger in patients with FS (Campbell et al., 2023), who were under-represented in this sample, although Mousa et al. (2021) also failed to show a significant correlation between dissociation and FS occurrence in their study. Our ongoing larger study will yield data on different types of dissociation in relation to the occurrence of FS and FMS, allowing us to explore possible differences between these groups.

#### 4.3.4 Autonomic arousal

Subjective physiological arousal and EMA-linked HR were both concurrently associated with FNS severity at the day-level. Subjective arousal was also correlated with FNS severity in the within-day concurrent analyses, although not in the combined/adjusted model. Subjective arousal, EMA-linked HR and EDA were not significant time-lagged predictors of FNS severity.

Elevated subjective arousal and HR, therefore, seem to be related to FNS severity at the day-level, but not necessarily on a moment-to-moment basis. These preliminary data do not definitively support previous laboratory-based findings suggesting that autonomic activation might be associated with the immediate onset or deterioration of momentary FNS (Pick et al., 2019; Pick, Millman et al., 2024); however, they suggest that increased autonomic arousal might have a more gradual, longer-term relationship to changes in FNS severity over the course of a day rather than from moment-to-moment, in naturalistic settings.

#### 4.3.5 Pain and fatigue

Pain and fatigue were correlated with FNS severity at the day- and within-day level and were also predictive of FNS severity in the within-day time-lagged model, although the latter was no longer significant in the combined model. This pattern of results illustrates that pain and fatigue covary with FNS severity on a day-to-day basis, and from moment-to-moment, which is compatible with studies describing elevated retrospective reports of pain and fatigue in FNSD (Butler et al., 2021; Ducroizet et al., 2023; Forejtova et al., 2023). Nonetheless, the available data do not support a particular direction of causal influence between pain, fatigue and FNS.

#### 4.3.6 Sleep disturbance

Objective sleep disturbance was associated with FNS severity at day-level, suggesting that FNS severity was greater on days when sleep disturbances were more numerous. This finding partially supports our hypotheses, but it is inconsistent with results presented by Mousa et al. (2021). The direction of the relationship between sleep disruption and FNS was unclear here because we did not provide evidence for a specific direction of temporal relationship between disturbed sleep and FNS ratings. The number of awakenings captured was also high relative to those observed in other studies (e.g., Thota, 2020); therefore, these may reflect partial arousals rather than full awakenings. Further data on relationships between sleep disturbance, duration and FNS are needed, including the use of research-grade wearables and/or polysomnography.

### 4.4 Objective and subjective markers of arousal and affect

There were inconsistent relationships between objective autonomic markers (EDA, HR) and subjective arousal and affect between groups. These results resembled the discrepant findings observed in laboratory and clinical settings (Adewusi et al., 2021; Pick et al., 2019), broadly suggesting that there may be divergent integration of subjective and objective markers of arousal and affect in FNSD (Adewusi et al., 2021; Pick, Mellers and Goldstein, 2016b; 2018a, 2024).

### 4.5 Strengths and limitations

This is the first study to combine EMA with wearables to examine a range of potential subjective and objective influences on FNS in patients’ daily lives. The findings provide new ecologically valid evidence supporting the possible role of daily events and negative affect in triggering or exacerbating FNS, in the short-term. Our data also underscore the presence of a constellation of psychobiological symptoms that covary with FNS, comprising elevated subjective arousal, pain, fatigue, dissociation, and negative affect. The study adopted a hypothesis-driven approach, controlled for multiple testing, and accounted for confounding variables, also providing evidence of patient acceptability and engagement with intensive RMT.

Nonetheless, the study was constrained by several factors. As a pilot investigation, the study had a limited sample size, was not powered to detect smaller effects of potential clinical interest, and type 2 errors may have occurred. Nevertheless, whilst this pilot sample was modest in size, the total number of ecological momentary assessments taken was aligned with common practice in EMA investigations (Wrzus and Neubauer, 2023) and the sample size was comparable to previous remote monitoring studies in FNSD (Section 1).

Another limitation was that a proportion of participants in both groups showed suboptimal compliance to the EMA protocol with >30% missing EMA data, so the results presented here should be interpreted cautiously. The lack of a clinical control group in this study prohibits definitive conclusions regarding the exclusivity of these findings to FNSD.

Our choice of wearable was based on unobtrusiveness and acceptability for longer-term use (Polhemus et al., 2020); however, the Fitbit Charge 5 is a consumer-grade device potentially limited in accuracy. For example, the minimum sampling rate for heartrate was five seconds, which may have compromised the accuracy of heartrate measures at some timepoints. Alternative consumer- or research-grade devices might be considered in future studies.

Some of our EMA items were unvalidated (e.g., daily events, FNS severity) and it is possible that participants’ responses were influenced by demand characteristics, although we strongly encouraged participants to respond accurately and honestly at every prompt.

Missing data rates were high for EDA and EMA-linked HR; therefore, the analyses of these variables should be considered as provisional. In addition, the 7-day sampling period may not have captured longer-term variability in FNS. Finally, whilst the level of compensation offered was deemed appropriate by the participants and our FNSD Patient and Carer Panel, due to the intensiveness of EMA data sampling, this compensation level may have inflated completion rates, and this may not generalise to other studies with other compensation arrangements.

## 5 Conclusions

Patients with FNSD found participation in this intensive RMT study to be acceptable, suggesting that a longer-term study is feasible. A cluster of psychobiological symptoms covaried with FNS in daily life, and we observed directional temporal relationships suggesting that emotionally salient events and negative affect might represent real-time triggers of FNS occurrence or worsening. We have now adapted this protocol in a larger-scale study comparing patients with FS to those with FMS as their primary symptom, as well as healthy and clinical controls. Improved evidence for the proximal causes and mechanisms underpinning FNS will facilitate the development of more effective, targeted interventions, such as those aiming to improve emotional awareness and regulation, particularly in the context of emotionally significant daily events.

## Funding

This paper represents independent research part-funded by the National Institute for Health and Care Research (NIHR) Biomedical Research Centre at South London and Maudsley NHS Foundation Trust and King's College London. The views expressed are those of the authors and not necessarily those of the NHS, the NIHR or the Department of Health and Social Care. SP and LSMM were supported by a 10.13039/501100000265Medical Research Council Career Development Award to SP [MR/V032771/1].

For the purposes of open access, the author has applied a Creative Commons Attribution (CC BY) licence to any Accepted Author Manuscript version arising from this submission.

## Data availability

The raw data will be made available on reasonable request.

## CRediT authorship contribution statement

Funding acquisition: SP, MH (equal), TC (supporting). Conceptualisation: SP. Design/methodology: SP (lead), MH, TC, LHG, ASD, MJE, TRN, JSW, AATSR, BS, MAM (supporting). Investigation: SP (lead: recruitment/screening/data collection), BS (supporting: screening). Data curation (processing/preparation): LSMM (lead), SP (supporting/supervision), JD (supporting). Formal data analysis: SP (lead), JH, LSMM, JD (supporting). Software: SP. Validation/Project administration: SP. Visualisation: JH, SP. Resources: SP, MH (equal), TC (supporting). Writing: SP (original draft/review and editing), MH, TC, JD, LHG, MAM, LSMM, TRN, ASD, MJE, JSW (review and editing).

## Declaration of competing interest

The authors report no competing interests.

## Appendix Supplementary materials

Image, application 1

Image, application 2

Image, application 3
