# Supplementary material for: Real-time biopsychosocial antecedents and correlates of functional neurological symptoms in daily life: A pilot remote monitoring technology study
Source: Psychiatry Res. 2024 Dec;342:116247. doi: 10.1016/j.psychres.2024.116247 (PMC11876104; doi:10.1016/j.psychres.2024.116247)
Supplement: Supplementary file 1 [file mmc1.docx]

**Supplementary Materials**

**Supplementary Table 1. Background questionnaire comparisons**

|  | **FNSD** **(n = 17)** | | **HC** **(n = 17)** | | **Statistical values** |
| --- | --- | --- | --- | --- | --- |
| ***TEC Impact: Mdn (IQR)*** | 13 | 10 | 8 | 11 | W=87.5, p=0.051, r=0.34 |
| ***TEC Total: Mdn (IQR*** | 4 | 5 | 2 | 3 | W=90, p=0.061, r=0.32 |
| ***AQ Total: M (SD)*** | 20.4 | 7.19 | 17.3 | 6.9 | t(31.95)=-1.26, p=0.11, g=0.44 |
| ***AQ Attention to Detail: M (SD)*** | 4.4 | 2.6 | 4.5 | 2.7 | t(31.93)=0.20, p=0.42, g=0.07 |
| ***AQ Attention Switching: M (SD)*** | 6.24 | 2.61 | 4.41 | 1.7 | t(27.48)=-2.41, p=0.01, g=0.81 |
| ***AQ Communication: Mdn (IQR)*** | 2 | 2 | 2 | 4 | W=146 , p=0.97, r=0.01 |
| ***AQ Imagination: Mdn (IQR)*** | 2 | 2 | 2 | 1 | W=140.5, p=0.90, r=0.02 |
| ***AQ Social Skills: Mdn (IQR)*** | 3 | 4 | 2 | 2 | W=95.5, p=0.09, r=0.29 |
| ***MDI Disengagement: M (SD)*** | 75.6 | 26.9 | 53.7 | 8.4 | t(19.06)=-3.20, **p=0.003**, g=1.04 |
| ***MDI Depersonalisation: Mdn (IQR)*** | 56 | 79 | 47 | 0 | W=68, **p<0.001**, r=0.58 |
| ***MDI Derealisation: Mdn (IQR)*** | 57 | 44 | 46 | 0 | W=76, **p=0.005**, r=0.49 |
| ***MDI Memory Disturbance: Mdn (IQR)*** | 58 | 31 | 52 | 7 | W=80, p=0.023, r=0.39 |
| ***MDI Emotional Constriction: Mdn (IQR)*** | 46 | 4 | 46 | 4 | W=134.5, p=0.70, r=0.07 |
| ***MDI Identity Dissociation: Mdn (IQR)*** | 47 | 0 | 47 | 0 | W=119, p=0.08, r=0.31 |
| ***SDQ-20 Somatoform Dissociation:***  ***Mdn (IQR)*** | 29 | 9 | 20 | 0 | W=14.5, **p<0.001**, r=0.79 |
| ***PHQ-15 Physical Symptoms: M SD)*** | 13.5 | 4.0 | 3.2 | 2.4 | t(26.37)=-8.98**, p<0.001**, g=3.01 |
| ***PHQ-9 Depression: Mdn (IQR)*** | 12 | 8 | 1 | 3 | W=16.5, **p<0.001**, r=0.76 |
| ***GAD-7: Mdn (IQR)*** | 8 | 8 | 2 | 4 | W=46, **p<0.001**, r=0.58 |
| ***TAS-20 Alexithymia: M (SD)*** | 53.5 | 10.2 | 42.0 | 9.9 | t(31.99)-3.32, **p=0.001**, g=1.11 |
| ***SF-36 Energy/Fatigue: M (SD)*** | 25.9 | 17.4 | 65.3 | 13.3 | t(29.90)=7.41, **p<0.001**, r=2.48 |
| ***SF-36 General Health: M (SD)*** | 34.7 | 19.2 | 74.1 | 14.5 | t(29.80)=6.76, **p<0.001**, r=2.26 |
| ***SF-36 Emotional Wellbeing: Mdn (IQR)*** | 60 | 24 | 84 | 16 | W=236.5, **p=0.002**, r=0.55 |
| ***SF-36 Pain: Mdn (IQR)*** | 35 | 25 | 100 | 22.5 | W=274.5, **p<0.001**, r=0.78 |
| ***SF-36 Physical Functioning: Mdn (IQR)*** | 40 | 25 | 95 | 5 | W=289, **p<0.001**, r=0.86 |
| ***SF-36 Role Limitations-Emotional:***  ***Mdn (IQR)*** | 0 | 100 | 100 | 33.3 | W=213, **p=0.011**, r=0.44 |
| ***SF-36 Role Limitations-Physical:***  ***Mdn (IQR)*** | 0 | 0 | 100 | 0 | W=276, **p<0.001**, r=0.84 |
| ***SF-36 Social Functioning: Mdn (IQR)*** | 37.5 | 37.5 | 100 | 25 | W=261.5, **p<0.001**, r=0.70 |
| ***WSAS: Mdn (IQR)*** | 25 | 8 | 1 | 4.5 | W=1**, p<0.001**, r=0.84 |

**Key:** AQ=Autism Spectrum Quotient (range: 0-50, higher scores indicate more autistic traits); GAD-7=Generalized Anxiety Disorder–7 (range: 0-21, higher scores indicate higher anxiety levels); IQR=interquartile range; M=mean; MDI=Multiscale Dissociation Inventory (subscales range: 44-170, higher scores indicate more psychological dissociative symptoms); PHQ-9=Patient Health Questionnaire–9 (range: 0-27, higher scores indicate greater depressive symptomatology); PHQ-15=Patient Health Questionnaire–15 (range: 0-30, higher scores indicate greater somatic symptoms); SD=standard deviation; SDQ-20=Somatoform Dissociation Questionnaire–20 (range: 20-100, higher scores indicate more bodily dissociative symptoms); SF-36=36-item Short Form survey (range: 0-100, higher scores indicate better health-related quality of life); TAS-20=Toronto Alexithymia Scale–20 (range: 20-100, higher scores indicate greater difficulty identifying, describing and experiencing emotions); TEC=Traumatic Experiences Checklist (total score range: 0-29, higher scores indicate more adverse events); WSAS=Work & Social Adjustment Scale (range: 0-40, higher scores indicate more severe impairment)

**References**

Bagby RM, Parker JD, Taylor GJ: The twenty-item Toronto Alexithymia Scale: I. item selection and cross-validation of the factor structure. J Psychosom Res 1994; 38:23–32

Baron-Cohen S, Wheelwright S, Skinner R, et al: The Autism-Spectrum Quotient (AQ): evidence from Asperger syndrome/high-function autism, males and females, scientists and mathematicians. J Autism Dev Disord 2001; 31:5–17

Briere J, Weathers FW, Runtz M: Is dissociation a multidimensional construct? Data from the Multiscale Dissociation Inventory. J Trauma Stress 2005; 18:221–231

Broadbent E, Petrie KJ, Main J, et al: The Brief Illness Perception Questionnaire. J Psychosom Res 2006; 60:631–637

Hays RD, Sherbourne CD, Mazel RM: The RAND 36-Item Health Survey 1.0. Health Econ 1993; 2:217–227

Kroenke K, Spitzer RL, Williams JB: The PHQ-9: validity of a brief depression severity measure. J Gen Intern Med 2001; 16:606–613

Kroenke K, Spitzer RL, Williams JB: The PHQ-15: validity of a new measure for evaluating the severity of somatic symptoms.Psychosom Med 2002; 64:258–266

Mundt JC, Marks IM, Shear MK: The Work and Social Adjustment Scale: a simple measure of impairment in functioning. Br J Psychiatry 2002; 180:461–464

Nijenhuis ERS, Van der Hart O, Kruger K: The psychometric characteristics of the Traumatic Experiences Checklist (TEC): first finding among psychiatric outpatients. Clin Psychol Psychother 2002; 9:200–210

Nijenhuis ER, Spinhoven P, Van Dyck R, et al: The development and psychometric characteristics of the Somatoform Dissociation Questionnaire (SDQ-20). J Nerv Ment Dis 1996; 184:688–694

Spitzer RL, Kroenke K, Williams JB, et al: A brief measure for assessing generalized anxiety disorder: the GAD-7. Arch Intern Med 2006; 166:1092–1

**Supplementary Table 2. Missing data and outlier rates by variable and group**

|  | **FNSD** | | **HC** | |
| --- | --- | --- | --- | --- |
|  | **% Missing** | **%**  **Outliers** | **% Missing** | **% Outliers** |
| **Subjective variables (EMA)**  **(FNSD=17, HC=17)** |  |  |  |  |
| *Subjective arousal* | 18.3 | 1.8 | 21.6 | 2.6 |
| *Pain* | 18.4 | 1.7 | 21.6 | 3.4 |
| *Fatigue* | 18.5 | 1.2 | 21.6 | 1.5 |
| *Dissociation* | 19 | 1.7 | 21.9 | 2.8 |
| *Positive affect* | 18.5 | 1.0 | 21.9 | 0.8 |
| *Negative affect* | 18.5 | 3.2 | 21.9 | 3.2 |
| *FNS Severity* | 18.1 | 1.8 | **-** | **-** |
| *FNSD Impact* | 18.2 | 1.7 | - | - |
| *Seizure count (n=6)* | 17.5 | 3.0 | - | - |
| *Sleep disturbance (daily)* | 1.7 | 0 | 0.8 | 0 |
| *Sleep duration (daily)* | 1.7 | 0 | 1.7 | 0 |
| **Objective variables (Fitbit)**  **(FNSD=15; HC=16)** |  |  |  |  |
| *EDA scans* | 41.2 | 0 | 34.3 | 0.7 |
| *Average resting HR (daily)* | 5.7 | 0 | 14.3 | 0 |
| *Average HR (daily)* | 6.7 | 0 | 0.9 | 0 |
| *EMA-linked HR* | 26 | 0.3 | 28 | 1.9 |
| *Physical activity (daily)* | 9.5 | 0 | 0.9 | 0 |
| *Sleep duration (daily)* | 9.5 | 0 | 1.8 | 0 |
| *Sleep disturbance (daily)* | 9.5 | 0 | 1.8 | 0 |
| *Sleep efficiency (daily)* | 9.5 | 0 | 1.8 | 6.3 |

**Key:** EDA=electrodermal activity; EMA=ecological momentary assessment; FNSD=functional neurological symptom disorder; HC=healthy controls; HR=heart-rate

**Supplementary Table 3. Benjamini-Hochberg corrections: Week-level group comparisons**

| **Variable** | **Unadjusted p-value** | **Rank** | **B-H critical value** |
| --- | --- | --- | --- |
| Subjective arousal | **<0.001** | 1 | 0.011 |
| Pain | **<0.001** | 2 | 0.021 |
| Fatigue | **<0.001** | 3 | 0.032 |
| Dissociation | **0.003** | 4 | 0.042 |
| Resting HR | **0.004** | 5 | 0.053 |
| Total events | **0.007** | 6 | 0.063 |
| Objective sleep disturbance (awakenings) | **0.013** | 7 | 0.074 |
| Objective sleep duration | **0.018** | 8 | 0.084 |
| Subjective sleep duration | **0.024** | 9 | 0.095 |
| EDA | **0.031** | 10 | 0.110 |
| Negative affect | **0.041** | 11 | 0.116 |
| Stressful events | **0.041** | 12 | 0.126 |
| Sleep efficiency | 0.102 | 13 | 0.137 |
| EMA-linked HR | 0.137 | 14 | 0.147 |
| Total HR | 0.138 | 15 | 0.158 |
| Positive affect | 0.231 | 16 | 0.168 |
| Physical activity | 0.32 | 17 | 0.179 |
| Pleasant events | 0.375 | 18 | 0.189 |
| Subjective sleep disturbance | 0.496 | 19 | 0.200 |

**Key:** B-H=Benjamini-Hochberg; EDA=electrodermal activity HR=heart-rate

p-values in **bold** remained significant following Benjamini-Hochberg correction

**Supplementary Table 4. Week-level scores by group: outliers removed**

|  | **FNSD**  **(n=17)** | | **HC**  **(n=17)** | | **Between-group comparisons** | | |
| --- | --- | --- | --- | --- | --- | --- | --- |
|  | **M**  **(SD)** | **Mdn (IQR)** | **M**  **(SD)** | **Mdn (IQR)** | **Test statistic** | **p-value** | **Effect size** |
| ***Arousal average***  *Outliers removed: FNSD=1* | **2.7 (0.9)** | **3.0 (1.7)** | 1.5 (0.7) | 1.2 (1.0) | U=37.0 | **<0.001** | **r=0.62** |
| ***Pain average***  *Outliers removed: HC=1* | 3.2 (1.4) | 3.2 (1.9) | **1.2 (0.3)** | **1.2 (0.4)** | **U=24.0** | **<0.001** | **r=0.70** |
| ***Dissociation average***  *Outliers removed: HC=1* | 1.9 (1.2) | 1.6 (1.7) | **1.0 (0.6)** | **1.0 (0.0)** | **U=53.0** | **0.002** | **r=0.52** |
| ***FNS severity average***  *Outliers removed: FNSD=1* | **2.9 (0.8)** | **2.7 (1.0)** | - | - | - | - | - |
| ***FND impact average***  *Outliers removed: FNSD=1* | **2.8 (0.6)** | **2.7 (0.6)** | - | - | - | - | - |
| ***Subjective sleep disturbance average***  *Outliers removed: HC=1* | 3.0 (1.0) | 3.1 (1.6) | **2.6 (0.8)** | **2.6 (0.9)** |  |  |  |
| ***Subjective sleep duration (hrs) average***  *Outliers removed: HC=1* | 7.3 (0.9) | 7.0 (1.6) | **6.6 (0.8)** | **6.6 (1.3)** |  |  |  |
| ***Objective sleep duration (mins) average***  *Outliers removed: FNSD=1* | **444.4 (44.8)** | **453.8 (63.9)** | 386.6 (55.6) | 395.4 (108.3) | **t(28)=**  **-3.11** | **0.002** | **g=1.11** |
| ***Total events***  *Outliers removed: FNSD=1* |  | **3.0 (3.8)** |  | 1.0 (2.0) | U=67.0 | **0.012** | **r=0.44** |
| ***Total stressful events***  *Outliers removed: FNSD=1* |  | **1.5 (3.0)** |  | 0.0 (1.0) | U=85.0 | **0.068** | **r=0.35** |
| ***Total pleasant events***  *Outliers removed: FNSD=1* |  | **0.0 (1.0)** |  | 0.0 (0.0) | U=118.0 | **0.533** | **r=0.15** |
| ***Sleep efficiency (%; minutes asleep/minutes in bed)***  *Outliers removed: HC=1* | 0.85  (0.06) | 0.87  (0.03) | **0.89**  **(0.02)** | **0.89**  **(0.02)** | U=65.0 | **0.051** | **r=0.36** |

**Key:** FNSD=functional neurological symptom disorder; HC=healthy controls; HR=heart-rate; IQR=interquartile range; M=mean; Mdn=median; SD=standard deviation

***Bold and underlined values amended following removal of outliers**

**Supplementary Table 5. Benjamini-Hochberg corrections: Day-level models**

| **Variable** | **Unadjusted p-value** | **Rank** | **B-H Critical Value** |
| --- | --- | --- | --- |
| ***Pain (concurrent)*** | **<0.001** | 1 | 0.005 |
| ***Fatigue (concurrent)*** | **<0.001** | 2 | 0.011 |
| ***Total events (concurrent)*** | **<0.001** | 3 | 0.017 |
| ***Negative affect (concurrent)*** | **0.001** | 4 | 0.022 |
| ***Subjective arousal (concurrent)*** | **0.002** | 5 | 0.028 |
| ***Positive affect (concurrent)*** | **0.002** | 6 | 0.033 |
| ***Stressful events (concurrent)*** | **0.006** | 7 | 0.039 |
| ***Pleasant events (concurrent)*** | **0.015** | 8 | 0.044 |
| ***Objective sleep disturbance (concurrent)*** | **0.033** | 9 | 0.050 |
| ***EMA-linked HR (concurrent)*** | **0.045** | 10 | 0.056 |
| ***Negative affect (concurrent)*** | 0.087 | 11 | 0.061 |
| ***Physical activity (lagged)*** | 0.117 | 12 | 0.067 |
| ***Objective sleep duration (concurrent)*** | 0.126 | 13 | 0.072 |
| ***Subjective sleep duration (lagged)*** | 0.206 | 14 | 0.078 |
| ***Dissociation (concurrent)*** | 0.277 | 15 | 0.083 |
| ***Subjective arousal (lagged)*** | 0.281 | 16 | 0.089 |
| ***Total HR (lagged)*** | 0.301 | 17 | 0.094 |
| ***Total events (lagged)*** | 0.319 | 18 | 0.100 |
| ***Pleasant events (lagged)*** | 0.354 | 19 | 0.110 |
| ***Subjective sleep disturbance (concurrent)*** | 0.36 | 20 | 0.111 |
| ***EDA (lagged)*** | 0.386 | 21 | 0.117 |
| ***Objective sleep disturbance (lagged)*** | 0.442 | 22 | 0.122 |
| ***Positive affect (lagged)*** | 0.443 | 23 | 0.128 |
| ***Pain (lagged)*** | 0.467 | 24 | 0.133 |
| ***Physical activity (concurrent)*** | 0.474 | 25 | 0.139 |
| ***Stressful events (lagged)*** | 0.496 | 26 | 0.144 |
| ***Dissociation (lagged)*** | 0.533 | 27 | 0.150 |
| ***Subjective sleep disturbance (lagged)*** | 0.568 | 28 | 0.156 |
| ***Objective sleep duration (lagged)*** | 0.574 | 29 | 0.161 |
| ***Resting HR (concurrent)*** | 0.608 | 30 | 0.167 |
| ***Fatigue (lagged)*** | 0.675 | 31 | 0.172 |
| ***Total HR (concurrent)*** | 0.74 | 32 | 0.178 |
| ***Resting HR (lagged)*** | 0.873 | 33 | 0.183 |
| ***EMA-linked HR (lagged)*** | 0.874 | 34 | 0.189 |
| ***EDA (concurrent)*** | 0.912 | 35 | 0.194 |
| ***Subjective sleep duration (concurrent)*** | 0.947 | 36 | 0.200 |

**Key:** B-H=Benjamini-Hochberg; EDA=electrodermal activity; EMA=ecological momentary assessment;

HR=heart-rate

p-values in **bold** remained significant following Benjamini-Hochberg correction

**Supplementary Table 6. Combined day-level concurrent predictors of FNS severity**

|  | **Unadjusted model** | | | | **Adjusted model** | | | |
| --- | --- | --- | --- | --- | --- | --- | --- | --- |
|  | ***ß*** | ***SE*** | **95% CI** | **p-value** | ***ß*** | ***SE*** | **95% CI** | **p-value** |
| ***Subjective arousal*** | 0.162 | 0.086 | -0.009 – 0.333 | 0.063 | 0.215 | 0.103 | 0.009 – 0.420 | **0.041** |
| ***Pain*** | 0.322 | 0.100 | 0.123 – 0.521 | **0.002** | 0.302 | 0.094 | 0.114 – 0.490 | **0.002** |
| ***Fatigue*** | 0.148 | 0.103 | -0.058 – 0.354 | 0.156 | 0.294 | 0.123 | 0.049 – 0.539 | **0.019** |
| ***Negative affect*** | 0.036 | 0.159 | -0.282 – 0.353 | 0.824 | 0.067 | 0.149 | -0.229 – 0.363 | 0.654 |
| ***Positive affect*** | -0.222 | 0.110 | -0.441 -  -0.004 | **0.046** | -0.177 | 1.000 | -0.376 –  0.021 | 0.080 |
| ***Total events*** | 0.226 | 0.065 | 0.097 – 0.354 | **<0.001** | 0.187 | 0.070 | 0.047 – 0.327 | **0.010** |
| ***EMA-linked HR*** | 0.017 | 0.010 | -0.003 – 0.037 | 0.093 | 0.018 | 0.009 | 0.000 – 0.036 | **0.048** |
| ***Objective sleep disturbance*** | 0.007 | 0.005 | -0.003 – 0.018 | 0.178 | 0.006 | 0.007 | -0.008 – 0.020 | 0.428 |
| ***Age*** | - | - | - | - | -0.020 | 0.012 | -0.045 – 0.005 | 0.118 |
| ***Gender*** | - | - | - | - | -0.680 | 0.352 | -1.38 – 0.020 | 0.057 |
| ***Mental health diagnosis*** | - | - | - | - | 0.198 | 0.214 | -0.229 – 0.624 | 0.359 |
| ***Physical health diagnosis*** | - | - | - | - | 0.213 | 0.291 | -0.365 – 0.792 | 0.465 |
| ***Baseline FNS severity*** | - | - | - | - | 0.880 | 0.093 | 0.695 – 1.07 | **<0.001** |
| **Model estimation** | | | | | | | | |
| **ICC (adjusted)** | 0.889 | | | | 0.617 | | | |
| **-2LL** | 162.8 | | | | 111.5 | | | |
| **AIC** | 184.8 | | | | 161.5 | | | |
| **BIC** | 212.5 | | | | 224.6 | | | |

***Key:*** AIC=Akaike’s Information Criterion; BIC=Bayesian Criterion; CI=confidence interval; HR=heartrate; ICC=intraclass correlation; -2LL=-2 log-likelihood; SE=standard error

p-values in **bold** were statistically significant

**Supplementary Table 7. Benjamini-Hochberg corrections: Within-day models**

| **Variable** | **Unadjusted p-value** | **Rank** | **B-H Critical Value** |
| --- | --- | --- | --- |
| ***Pain (concurrent)*** | **<0.001** | 1 | 0.009 |
| ***Fatigue (concurrent)*** | **<0.001** | 2 | 0.018 |
| ***Stressful events (concurrent)*** | **<0.001** | 3 | 0.027 |
| ***Negative affect (concurrent)*** | **0.001** | 4 | 0.036 |
| ***Positive affect (concurrent)*** | **0.002** | 5 | 0.045 |
| ***Total events (concurrent)*** | **0.002** | 6 | 0.054 |
| ***Total events (lagged)*** | **0.002** | 7 | 0.064 |
| ***Subjective arousal (concurrent)*** | **0.004** | 8 | 0.073 |
| ***Negative affect (lagged)*** | **0.006** | 9 | 0.082 |
| ***Fatigue (lagged)*** | **0.011** | 10 | 0.091 |
| ***Dissociation (lagged)*** | **0.016** | 11 | 0.100 |
| ***Pain (lagged)*** | **0.023** | 12 | 0.109 |
| ***Positive affect (lagged)*** | **0.037** | 13 | 0.118 |
| ***Stressful events (lagged)*** | 0.051 | 14 | 0.127 |
| ***Subjective arousal (lagged)*** | 0.103 | 15 | 0.136 |
| ***Pleasant events (lagged)*** | 0.123 | 16 | 0.145 |
| ***Dissociation (concurrent)*** | 0.167 | 17 | 0.154 |
| ***EMA-linked HR (lagged)*** | 0.174 | 18 | 0.164 |
| ***Pleasant events (concurrent)*** | 0.635 | 19 | 0.173 |
| ***EDA (concurrent)*** | 0.75 | 20 | 0.182 |
| ***EMA-linked HR (concurrent)*** | 0.767 | 21 | 0.191 |
| ***EDA (lagged)*** | 0.931 | 22 | 0.200 |

**Key:** B-H=Benjamini-Hochberg; EDA=electrodermal activity; EMA=ecological momentary assessment; HR=heart-rate

p-values in **bold** remained significant following Benjamini-Hochberg correction

**Supplementary Table 8. Combined concurrent predictors of momentary FNS severity**

|  | **Unadjusted model** | | | | **Adjusted model** | | | |
| --- | --- | --- | --- | --- | --- | --- | --- | --- |
|  | ***ß*** | ***SE*** | **95% CI** | **p-value** | ***ß*** | ***SE*** | **95% CI** | **p-value** |
| ***Subjective arousal*** | 0.108 | 0.057 | -0.004 – 0.220 | 0.059 | 0.131 | 0.070 | -0.007 – 0.270 | 0.063 |
| ***Pain*** | 0.325 | 0.076 | 0.174 – 0.474 | **<0.001** | 0.301 | 0.095 | 0.114 – 0.488 | **0.002** |
| ***Fatigue*** | 0.158 | 0.046 | 0.068 – 0.248 | **<0.001** | 0.101 | 0.050 | 0.003 – 0.200 | **0.044** |
| ***Positive affect*** | -0.104 | 0.046 | -0.194 - -0.014 | **0.023** | -0.052 | 0.052 | -0.155 – 0.051 | 0.322 |
| ***Negative affect*** | 0.084 | 0.079 | -0.072 – 0.240 | 0.290 | 0.250 | 0.088 | 0.077 – 0.424 | **0.005** |
| ***Total events*** | 0.609 | 0.171 | 0.273 – 0.945 | **<0.001** | 0.547 | 0.202 | 0.152 – 0.943 | **0.007** |
| ***Stressful events*** | -0.045 | 0.211 | -0.460 – 0.369 | 0.830 | -0.100 | 0.244 | -0.579 – 0.378 | 0.681 |
| ***Age*** | - | - | - | - | -0.036 | 0.022 | -0.080 – 0.007 | 0.102 |
| ***Gender*** | - | - | - | - | -2.08 | 0.579 | -3.21 – -0.939 | **<0.001** |
| ***Mental health diagnosis*** | - | - | - | - | -1.03 | 0.228 | -1.48 – -0.582 | **<0.001** |
| ***Physical health diagnosis*** | - | - | - | - | -0.774 | 0.361 | -1.48 – -0.064 | **0.033** |
| ***Baseline FNS severity*** | - | - | - | - | 0.737 | 0.090 | 0.560 – 0.915 | **<0.001** |
| **Model estimation** | | | | | | | | |
| **ICC (adjusted)** | 0.670 | | | | 0.175 | | | |
| **-2LL** | 2127.9 | | | | 1451.4 | | | |
| **AIC** | 2163.9 | | | | 1499.4 | | | |
| **BIC** | 2247.7 | | | | 1603.2 | | | |

***Key:*** AIC=Akaike’s Information Criterion; BIC=Bayesian Criterion; CI=confidence interval; ICC=intraclass correlation;

-2LL=-2 log-likelihood; SE=standard error

p-values in **bold** remained significant following Benjamini-Hochberg correction

**Supplementary Table 9. Combined time-lagged predictors of momentary FNS severity**

|  | **Unadjusted model** | | | | **Adjusted model** | | | |
| --- | --- | --- | --- | --- | --- | --- | --- | --- |
|  | ***ß*** | ***SE*** | **95% CI** | **p-value** | ***ß*** | ***SE*** | **95% CI** | **p-value** |
| ***Pain*** | 1.55 | 0.089 | -0.019 – 0.330 | 0.081 | 0.147 | 0.090 | -0.030 – 0.324 | 0.104 |
| ***Fatigue*** | 0.026 | 0.061 | -0.093 – 0.145 | 0.665 | -0.036 | 0.085 | -0.203 – 0.130 | 0.668 |
| ***Dissociation*** | 0.111 | 0.069 | -0.024 – 0.246 | 0.106 | 0.180 | 0.098 | -0.012 – 0.372 | 0.066 |
| ***Positive affect*** | 0.004 | 0.065 | -0.124 | 0.132 | 0.001 | 0.088 | -0.171 – 0.173 | 0.990 |
| ***Negative affect*** | 0.081 | 0.124 | -0.163 – 0.324 | 0.515 | 0.239 | 0.193 | 0.016 – 0.461 | **0.035** |
| ***Total events*** | 0.266 | 0.222 | -0.169 – 0.702 | 0.230 | 0.146 | 0.275 | -0.394 – 0.687 | 0.595 |
| ***Age*** | - | - | - | - | -0.048 | 0.024 | -0.096 – 0.000 | **0.048** |
| ***Gender*** | - | - | - | - | -2.38 | 0.636 | -3.63 - -1.13 | **<0.001** |
| ***Mental health diagnosis*** | - | - | - | - | -1.23 | 0.250 | -1.72 - -0.743 | **<0.001** |
| ***Physical health diagnosis*** | - | - | - | - | -1.07 | 0.400 | -1.86 - -0.283 | **0.008** |
| ***Baseline FNS severity*** | - | - | - | - | 0.791 | 1.00 | 0.594 – 0.988 | **<0.001** |
| **Model estimation** | | | | | | | | |
| **ICC (adjusted)** | 0.567 | | | | 0.116 | | | |
| **-2LL** | 1851.3 | | | | 1231.9 | | | |
| **AIC** | 1883.3 | | | | 1273.9 | | | |
| **BIC** | 1953.3 | | | | 1358.7 | | | |

***Key:*** AIC=Akaike’s Information Criterion; BIC=Bayesian Criterion; CI=confidence interval; ICC=intraclass correlation;

-2LL=-2 log-likelihood; SE=standard error

p-values in **bold** remained significant following Benjamini-Hochberg correction
