# Supplementary figures and images for: Real-time biopsychosocial antecedents and correlates of functional neurological symptoms in daily life: A pilot remote monitoring technology study
Source: Psychiatry Res. 2024 Dec;342:116247. doi: 10.1016/j.psychres.2024.116247 (PMC11876104; doi:10.1016/j.psychres.2024.116247)

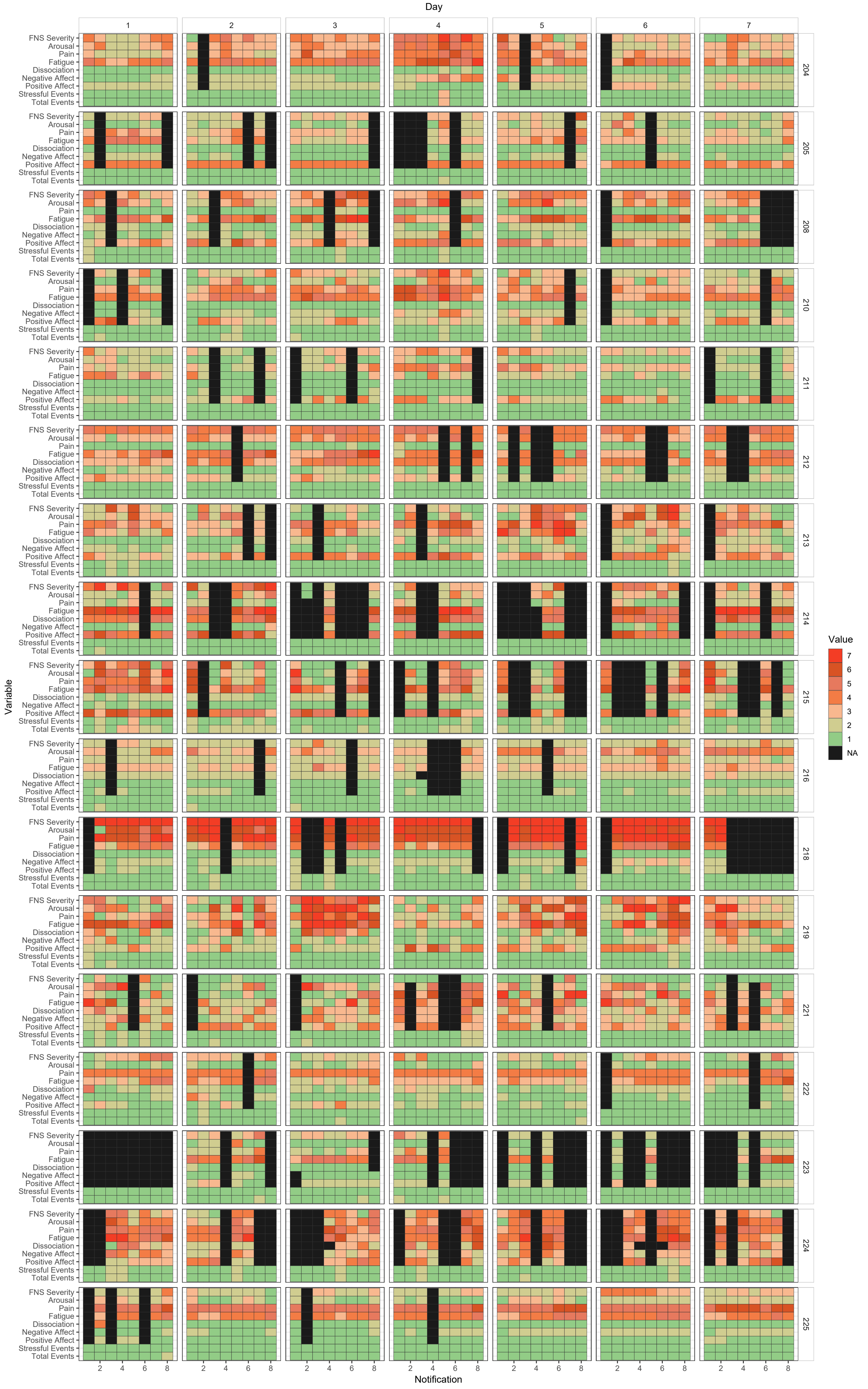

Supplement: Supplementary file 2 [file mmc2.pdf]

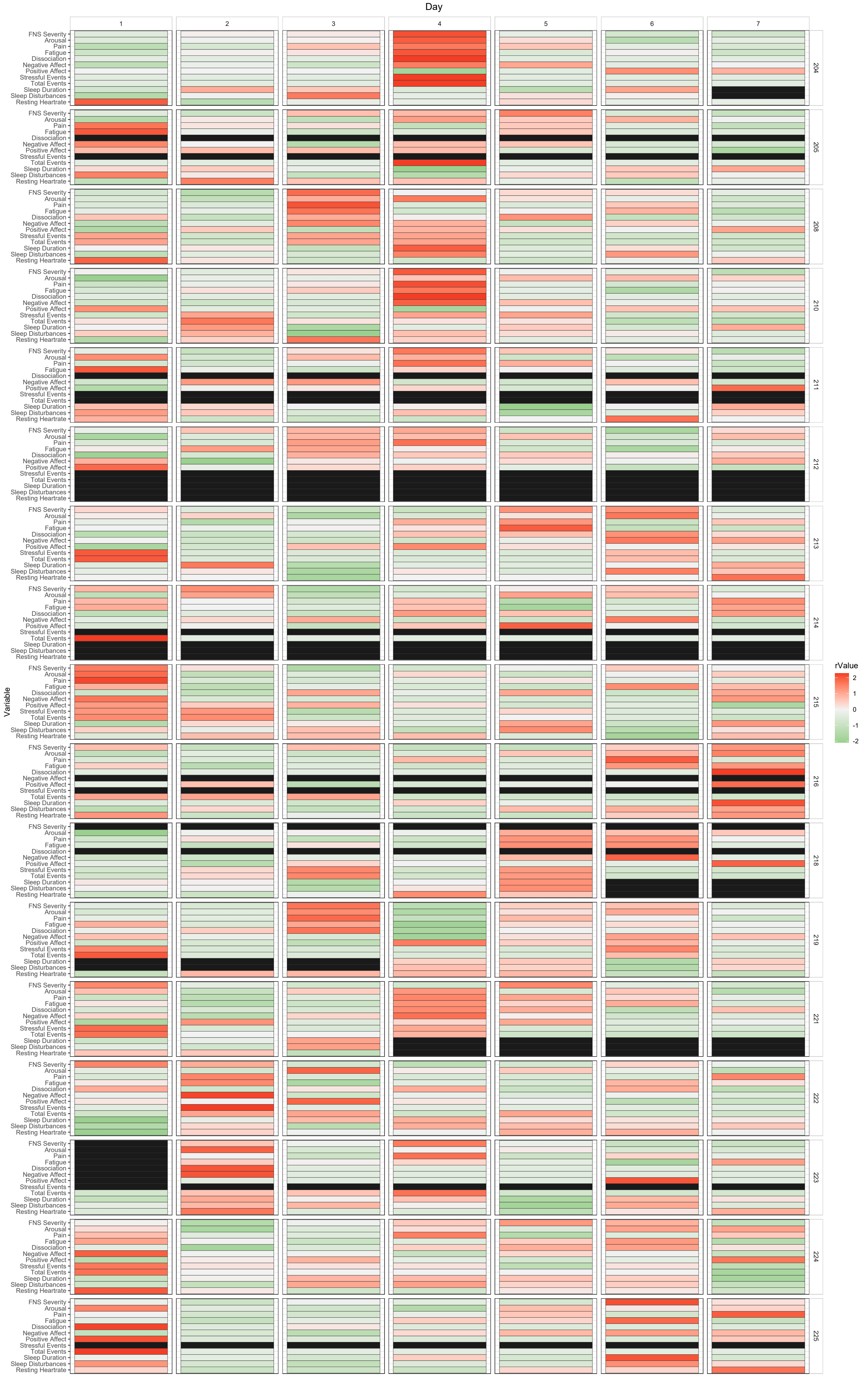

Supplement: Supplementary file 3 [file mmc3.pdf]
